# Gynecoplastic Surgery: A Unified Terminology for Female Genital Aesthetic, Reconstructive, and Functional Procedures

**DOI:** 10.7759/cureus.101015

**Published:** 2026-01-07

**Authors:** Jose M Togo, Thania M Hurtado, Pablo Gonzalez

**Affiliations:** 1 Laser Gynecology, Central Medica Quirurgica, Mazatlan, MEX; 2 Aesthetic Medicine, Central Medica Quirurgica, Mazatlan, MEX; 3 Gynecology, Asociación de Ginecólogos Estéticos Colombianos, Pereira, COL

**Keywords:** aesthetic genital surgery, functional gynecology, gynecoplastic surgery, labiaplasty, laser surgery, perineoplasty, vaginoplasty, vhi, vuhi, vulvovaginal aging

## Abstract

The field of female genital aesthetic, reconstructive, and functional surgery has rapidly expanded due to advances in laser and regenerative technology, but its fragmented terminology currently limits academic cohesion and standardization. This narrative review proposes gynecoplastic surgery as a unified term that integrates the aesthetic, reconstructive, and functional domains of female genital surgery. The review was structured using PubMed, Scopus, and Web of Science to consolidate knowledge on terminology, anatomical classification, and objective evaluation tools, including validated indices, such as the Vaginal Health Index and Vulvar Health Index, and imaging modalities, such as high-frequency ultrasound. The literature confirms a convergence of restorative, aesthetic, and functional procedural goals, supported by emerging objective metrics for vulvovaginal evaluation. Gynecoplastic surgery offers an encompassing framework, connecting the historical evolution of reconstructive gynecology with advances in imaging and regenerative therapies. Standardizing terminology under this framework will promote academic clarity, facilitate multidisciplinary collaboration, and align clinical practice with modern evidence-based assessment, supporting its future adoption in research, training, and patient communication.

## Introduction and background

The field of female genital aesthetic, reconstructive, and functional surgery has experienced sustained growth in recent years, driven by advances in energy-based technologies, improved anatomical understanding, and greater cultural acceptance of genital health and aesthetics as part of women’s overall well-being. However, this rapid expansion, often described as the globally rising tide of cosmetic gynecology, has also raised significant ethical concerns regarding misleading marketing, lack of long-term evidence, and insufficient professional regulation [1].

The international debate surrounding female genital cosmetic procedures has been deeply influenced by the ethical frameworks established by leading professional organizations. The Royal College of Obstetricians and Gynaecologists (RCOG) first addressed the issue in 2013, warning that many of these procedures are performed in the absence of clear medical indications, standardized training, or evidence-based outcomes. The RCOG emphasized that women must be fully informed of the wide range of normal genital variation, the potential for physical and psychological harm, and the lack of proven long-term benefits before consenting to any intervention [2].

Similarly, the American College of Obstetricians and Gynecologists (ACOG) reaffirmed this stance in its 2020 Committee Opinion, classifying female genital cosmetic surgery as nontherapeutic and emphasizing that physicians have an ethical obligation to provide unbiased counseling and avoid misrepresenting these procedures as routine medical treatments [3].

Building upon these positions, the International Federation of Gynecology and Obstetrics (FIGO) issued a restrictive statement in 2025 [4]. FIGO explicitly maintains that it is ethically inappropriate for obstetrician-gynecologists to recommend, perform, or refer patients for female genital cosmetic procedures, citing a profound lack of robust safety data and evidence-based outcomes, particularly concerning energy-based devices. The federation urged professional bodies to prioritize patient safety and discourage nonevidence-based practices driven by social or commercial pressure. This cautionary international stance underscores the urgent necessity for the terminological precision and academic framework that gynecoplastic surgery aims to establish, providing a scientific platform to address the very gaps in evidence and regulation identified by FIGO.

A compelling model for this unification is the historical evolution of oncoplastic breast surgery. In the early 1990s, Audretsch introduced the concept of combining oncologic safety with reconstructive and aesthetic principles, allowing surgeons to achieve complete tumor excision without compromising the cosmetic integrity of the breast [5]. This innovation marked the beginning of a paradigm shift in surgical oncology, demonstrating that function, form, and aesthetics could coexist under a single, ethically grounded framework. Subsequent studies by Clough et al. and Kaufman expanded this concept by integrating reconstructive design directly into the oncologic planning phase, creating a multidisciplinary approach that standardized techniques, improved patient satisfaction, and redefined surgical education [6,7].

Consequently, the term oncoplastic rapidly consolidated its position as a legitimate academic designation, representing the successful integration of therapeutic radicality with aesthetic refinement and comprehensive psychosocial recovery [8]. The formal recognition of oncoplastic breast surgery transformed an unregulated set of reconstructive techniques into a codified subspecialty. This precedent illustrates how a well-defined terminology can elevate a clinical practice from fragmented innovation to a standardized academic discipline. The consolidation of oncoplastic breast surgery serves as a historical precedent for the emerging field of female genital surgery.

Over the past two decades, a parallel transformation has been observed in gynecology, where increasing numbers of publications have described procedures addressing vulvovaginal form and function under various terminologies such as female genital cosmetic surgery, aesthetic vulvar surgery, and functional gynecology [9-15].

Goodman [9] was among the first to define and systematize the field of female genital aesthetic and plastic surgery, outlining its procedural diversity and clinical rationale while emphasizing the absence of standardized terminology and long-term data. Iglesia et al. [10] published the first comprehensive, peer-reviewed overview of female genital cosmetic surgery in a urogynecologic journal, systematically describing techniques, outcomes, and complication profiles across more than 700 reported procedures. Their review clarified that the boundary between cosmetic and reconstructive intent is inherently fluid, as many operations pursue both aesthetic refinement and functional restoration. Beyond surgical detail, the authors emphasized essential ethical and psychological safeguards: thorough patient education about normal genital variation, exclusion of body dysmorphic tendencies, avoidance of coercion or marketing bias, and the surgeon's responsibility to obtain truly informed consent. This publication consolidated the transition of genital aesthetic surgery from anecdotal practice to structured clinical discourse, bridging gynecology, urogynecology, and plastic surgery under a shared scientific framework.

Hamori et al. [11] reframed genital aesthetic surgery as a practice that frequently improves comfort, symmetry, and self-image, demonstrating the intrinsic connection between functional and aesthetic results. Sorice et al. [12] and Clerico et al. [13] broadened this understanding by documenting patient motivations and anatomical variability, showing that most women pursue these procedures to relieve discomfort or self-consciousness rather than to meet external ideals. Finally, Placik and Devgan [14] proposed a multidisciplinary framework integrating reconstructive design, aesthetic harmony, and psychosocial well-being, thereby consolidating the modern scientific identity of the field.

Collectively, these publications established the intellectual and clinical foundation for viewing female genital surgery as a domain where anatomy, function, and perception converge. Yet, despite the proliferation of techniques and devices, the lack of standardized terminology and consistent ethical oversight has remained a persistent challenge. The International Urogynecological Association (IUGA) and the American Urogynecologic Society (AUGS) addressed this gap in their 2022 joint terminology report, recognizing that "cosmetic gynecology" had become an umbrella term encompassing heterogeneous procedures performed under variable indications and with inconsistent training requirements [15]. The document emphasized the need for clear, anatomically based terminology that differentiates aesthetic goals from reconstructive or therapeutic interventions while acknowledging their frequent overlap. This position represents a milestone in legitimizing the field through linguistic precision and ethical alignment.

Building upon this framework, several authors have contributed essential anatomical classifications that delineate the structural domains of the external genital region and form the foundation of gynecoplastic surgery.

Crucially, the essential anatomical classifications that delineate the structural domains of the external genital region, foundational to the concept of gynecoplastic surgery, were previously contributed by several authors, providing the necessary clinical basis for this unified framework. El-Khatib established the first surgical classification of mons pubis ptosis, analyzing 132 women undergoing abdominoplasty with concurrent pubic lift. He categorized deformities into four progressive grades based on fat volume, cutaneous excess, and coverage of the genital cleft: Grade I (mild bulging, no coverage), managed with liposuction; Grade II (moderate projection, partial vulvar coverage), treated by lipectomy assisted with liposuction; Grade III (marked protrusion, complete vulvar coverage), requiring combined lipectomy and dermofascial suspension; and Grade IV (severe descent without bulging, typical of post-bariatric patients), corrected with vertical resection and fascial fixation. This algorithmic approach defined the adipose compartment of the vulvoperineal unit, emphasizing fascial suspension to restore pubic projection and aesthetic harmony without displacing the urethral meatus or clitoral position. The classification remains the reference standard for integrating mons correction into composite gynecoplastic or abdominoplasty procedures [16].

Focusing on the labia majora compartment, Fasola and Gazzola provided the first clinical grading for labia majora hypotrophy, based on the treatment of 54 women with hyaluronic acid (HA) filler. They identified three degrees of severity based on cutaneous texture, subcutaneous volume, and symptoms of atrophy: Grade I (mild): subtle subcutaneous thinning and fine wrinkling, generally asymptomatic or associated with minor post-weight-loss changes; Grade II (moderate): moderate loss of turgor with visible laxity, dryness, and occasional discomfort or dyspareunia; Grade III (severe): pronounced deflation, deep wrinkling, and clear signs of vulvar atrophy. Their work provided a reproducible protocol for restoring the labia majora compartment, highlighting the anatomical safety of the inter-dartos plane and the reversibility of the filler as advantages for both functional comfort and aesthetic rejuvenation [17].

Palacios [18] provided a pivotal redefinition of Vaginal Hyperlaxity Syndrome, characterizing it as a functional pathology of the pelvic connective tissues rather than a purely cosmetic concern. He established a syndromic model to differentiate physiological laxity from true hyperlaxity, combining anatomical parameters (genital hiatus, vaginal wall distensibility, and pelvic organ prolapse quantification) with clinical findings such as decreased friction, vaginal flatus, or mild incontinence. Furthermore, Palacios established a three-tiered management system based on severity: mild laxity management involves pelvic-floor exercises for muscular strengthening. Moderate grades utilize energy-based therapies (CO_2_ or erbium-doped yttrium aluminum garnet laser, radiofrequency) to induce collagen remodeling and neovascularization. Severe hyperlaxity requires vaginoperineoplasty to restore muscular continuity and perineal body support.

This comprehensive contribution firmly established the vaginal compartment as a functional unit with objective diagnostic criteria and evidence-based therapeutic algorithms, successfully transforming the ambiguous notion of "vaginal rejuvenation" into a medically defined syndrome.

Completing this anatomical progression, González-Isaza and Sánchez-Borrego [19] provided the most comprehensive topographic framework to date. Synthesizing prior insights from the work by González-Isaza and Sánchez-Borrego [19], their work established a detailed organization of the vulva into four interdependent compartments: epithelial, fascial, erectile, and adipose. Central to this model is a quantitative hypertrophy scale for the labia minora based on projection beyond the labia majora: Grade I: ≤ 1 cm; Grade II: 1-3 cm; Grade III: 3-5 cm; Grade IV: > 5 cm. This rigorous classification unifies morphological and functional considerations, enabling precise and reproducible planning for labia minora reduction or contouring procedures [19].

Rodas et al. [20] recently proposed a novel Integrative Grading Scale for Vulvovaginal Aging. This comprehensive assessment tool combines clinical, imaging, and symptom-based parameters. The system incorporates validated metrics, such as the Vaginal Health Index (VHI) and Vulvar Health Index (VuHI), alongside imaging modalities, including three-dimensional photogrammetry and high-frequency ultrasound, to objectively quantify tissue changes and treatment outcomes. By integrating morphological assessment with functional evaluation, Rodas et al. established a model of quantifiable precision that aligns seamlessly with the conceptual framework of gynecoplastic surgery.

Collectively, the sequential contributions of El-Khatib [16], Fasola and Gazzola [17], Palacios [18], and González-Isaza and Sánchez-Borrego [19] define the four principal anatomical compartments, mons pubis, labia majora, vagina, and labia minora, that constitute the structural and conceptual backbone of gynecoplastic surgery. These classifications culminate in a unified topographic system that rigorously aligns aesthetic, reconstructive, and functional principles within an ethical and scientific framework.

Together, the contributions establishing anatomical categorization, ethical oversight (IUGA) [15], and objective diagnostic tools [20] form the three pillars required to elevate female genital surgery into a mature scientific discipline. Gynecoplastic surgery, therefore, represents the logical and necessary synthesis of these efforts, a unified terminology that integrates aesthetic refinement, functional restoration, and ethical accountability under a single academic identity.

## Review

Materials and methods

This study was conducted as a structured narrative review aimed at consolidating and critically analyzing current knowledge regarding terminology, anatomical classifications, and objective assessment tools within female genital aesthetic, reconstructive, and functional surgery. A comprehensive search was performed in PubMed, Scopus, and Web of Science from January 2011 to October 25, 2025 (the date of the final search). The search utilized the following complete string: ("female genital surgery" OR "cosmetic gynecology" OR "aesthetic vulvar surgery" OR "reconstructive gynecology" OR "laser vaginal rejuvenation") AND ("functional outcomes" OR "classification" OR "terminology"). Additional manual searches were carried out in reference lists of relevant reviews, textbooks, and institutional guidelines to ensure the inclusion of both seminal and recent works in English and Spanish.

Selection Process

Initial identification yielded 142 records. After removing duplicates and screening titles and abstracts, 54 full-text articles were assessed for eligibility. Inclusion criteria encompassed publications addressing the evolution of terminology, the development of anatomical and procedural classification systems, and the integration of validated clinical indices such as the VHI, VuHI, and patient-reported outcomes (Female Sexual Function Index (FSFI) and Day-to-Day Impact of Vaginal Aging (DIVA)). Studies describing objective imaging modalities, including high-frequency ultrasound and three-dimensional photogrammetry, were also included. Articles focusing exclusively on pediatric populations or male genital surgery were excluded. Purely oncologic procedures lacking reconstructive or aesthetic components were also excluded; however, seminal works on oncoplastic breast surgery were included as foundational conceptual models for integrating aesthetic and reconstructive principles. Finally, 26 key sources were selected for qualitative synthesis based on their contribution to the gynecoplastic surgery framework.

Data Synthesis and Quality Assessment

Data extraction emphasized the convergence between anatomical restoration, aesthetic refinement, and functional enhancement. The findings were synthesized into three major analytical categories: historical fragmentation, the anatomical domains of gynecoplastic surgery, and emerging diagnostic tools. Although a formal Grading of Recommendations Assessment, Development, and Evaluation evidence profile was not conducted due to the narrative and foundational nature of this review, each source was qualitatively appraised for clinical relevance and internal consistency. We acknowledge an inherent risk of bias, as current literature in this field predominantly consists of descriptive studies and expert opinions rather than long-term randomized controlled trials (RCTs). This integrative approach establishes a structured academic framework intended to align clinical practice and standardized terminology within a single scientific discipline.

Results

Conceptual Integration of Gynecoplastic Surgery

**Figure 1:**
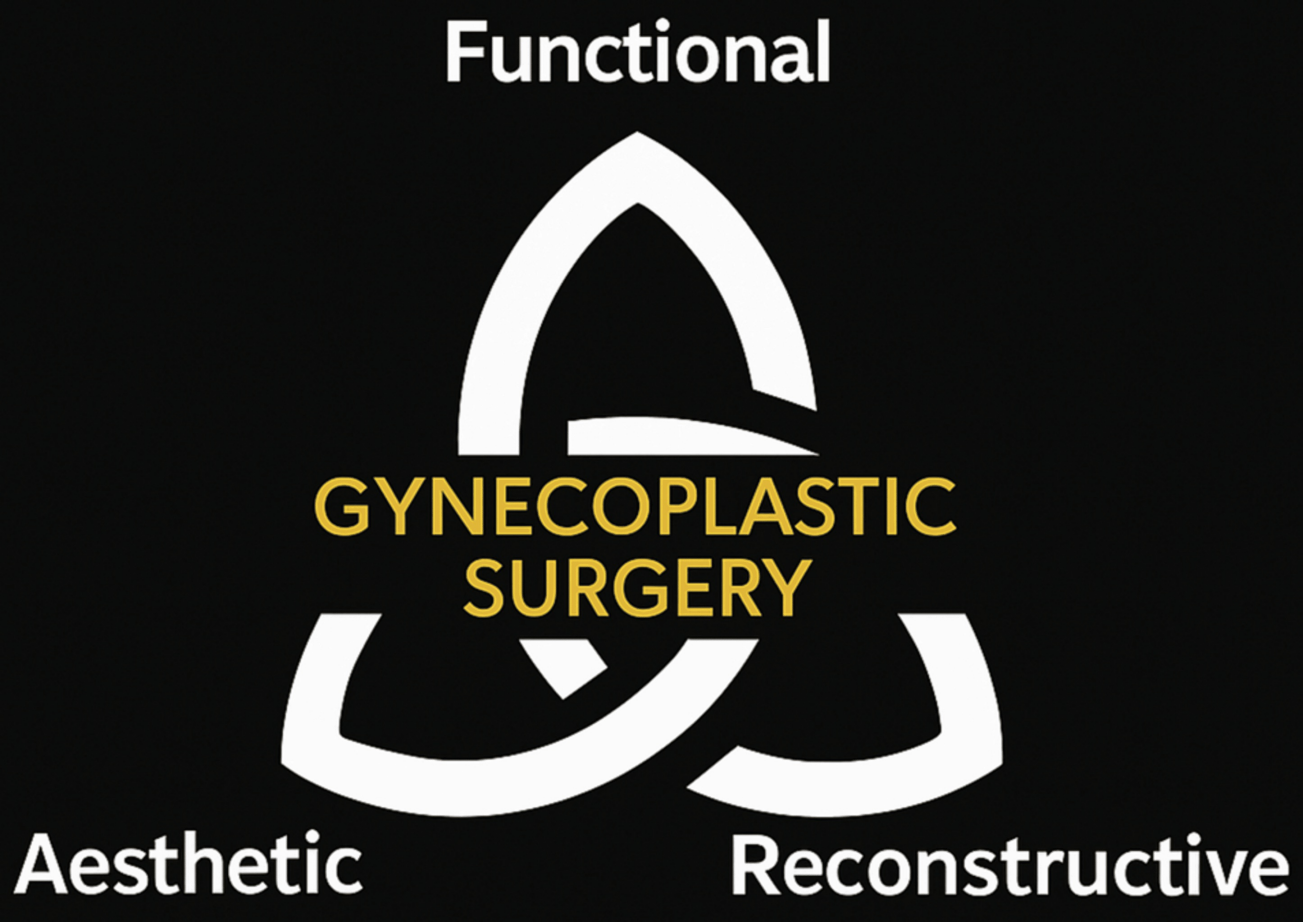
Conceptual structure of gynecoplastic surgery The three interconnected domains, Aesthetic, Reconstructive, and Functional, represent the unified foundation of the discipline Image credit: This is an original image created by the author Jose M. Togo Sr.

The reviewed literature suggests a tendency toward convergence between aesthetic, reconstructive, and functional approaches to female genital surgery. Initially conceived as separate goals, these dimensions have progressively merged through advances in anatomical understanding, regenerative science, and minimally invasive technology (Figure 1). The concept of gynecoplastic surgery emerges as a unifying framework that recognizes this interdependence and situates genital surgery within an evidence-based and ethically defined discipline (Table 1). This integration mirrors the evolution of oncoplastic breast surgery, where reconstructive design became integral to oncologic treatment. In a similar manner, gynecoplastic surgery replaces fragmented terms such as cosmetic gynecology, aesthetic vulvar surgery, or functional gynecology with a single descriptor that reflects its scientific coherence, anatomical precision, and ethical responsibility.

**Table 1:** Conceptual domains of gynecoplastic surgery This framework integrates anatomical, functional, aesthetic, regenerative, and sensory dimensions within a single surgical philosophy. Each domain represents an interdependent component of female genital restoration and performance Sensory and neurosensory enhancement objectives are reported in the literature as clinical hypotheses; however, they require further validation through neurophysiological studies and robust patient-reported outcomes to establish definitive efficacy Source: Adapted from the academic and clinical perspectives described in [9-11,14,19,20] and consistent with the unified terminology proposed by the International Urogynecological Association and the American Urogynecologic Society (2022) [15]

Domain	Core objective	Primary clinical impact
Anatomical restoration	Reconstruct or reinforce vulvovaginal structures compromised by trauma, childbirth, congenital variation, or oncologic surgery	Reestablished support, symmetry, and structural integrity; improved comfort and self-perception
Functional optimization	Enhance tone, lubrication, coaptation, and neurosensory response through surgical or regenerative interventions	Improved sexual response, urinary control, and vaginal health
Aesthetic-structural harmonization	Achieve proportional, symmetrical, and age-consistent contour while preserving or improving functionality	Balanced vulvovaginal appearance, reduction of irritation, and improved confidence and body image
Regenerative-restorative domain	Reverse aging-related atrophy, laxity, and dyschromia through biostimulation and tissue renewal	Enhanced mucosal trophism, elasticity, and color uniformity
Neurosensory enhancement	Optimize clitoral and vestibular sensitivity by improving tissue exposure and neural feedback	Potential for amplified arousal and orgasmic response

Anatomical and Procedural Framework

Across the analyzed sources, four major anatomical domains consistently define the surgical field: epithelial, fascial, erectile, and adipose compartments. This topographic model supports reproducibility, surgical planning, and interdisciplinary communication. Within this structure, several authors have contributed detailed classifications that clarify the anatomical boundaries and therapeutic strategies of each compartment.

The adipose compartment was first systematized through the classification of mons pubis ptosis, which grades the degree of adipose descent and genital coverage and defines fascial suspension techniques to restore natural contour and projection. Subsequent work on the labia majora compartment established a three-grade system for hypotrophy based on dermal thinning, volume loss, and associated symptoms such as dryness or dyspareunia, providing an injectable treatment protocol using HA in two planes of infiltration for aesthetic and functional restoration.

The vaginal compartment was later redefined as a functional structure rather than an aesthetic target, introducing the concept of vaginal hyperlaxity as a connective-tissue disorder characterized by reduced friction and pelvic support. This model outlined severity-based management options ranging from pelvic-floor reeducation to energy-based modalities and reconstructive surgery, establishing objective parameters for diagnosis and treatment selection.

Finally, the vulvar compartment was organized through the topographic classification of labia minora hypertrophy, dividing the vulva into four interdependent layers: epithelial, fascial, erectile, and adipose, and grading projection beyond the labia majora. This system integrates morphology, symmetry, and tissue dynamics, allowing standardized communication and reproducible outcomes.

Together, these anatomical frameworks delineate the structural foundation of gynecoplastic surgery, linking each procedure to a specific compartmental focus: labiaplasty (epithelial and fascial), clitoral hood contouring (erectile), perineoplasty and vaginoplasty (fascial and mucosal), labia majora augmentation (adipose), and mons correction (adipose and fascial). Grouping these interventions within a single conceptual system promotes uniform terminology and facilitates collaboration among gynecologists, urogynecologists, and plastic surgeons. It also provides the basis for competency-based training programs and standardized reporting of outcomes.

Objective Evaluation and Diagnostic Tools

A notable shift in recent literature involves the incorporation of objective and patient-reported assessment tools to evaluate outcomes beyond subjective satisfaction. The VHI and VuHI allow reproducible assessment of mucosal integrity, lubrication, and elasticity. Patient-reported measures such as the FSFI and the DIVA questionnaire quantify the functional and psychosocial dimensions of genital health (Table 2). Imaging technologies, particularly high-frequency ultrasound, transperineal ultrasound, and three-dimensional photogrammetry, provide measurable data on tissue architecture, thickness, and volumetric change. These modalities contribute to greater scientific rigor in documenting procedural efficacy and long-term tissue behavior, reinforcing the shift toward evidence-based genital surgery.

**Table 2:** Objective assessment tools relevant to gynecoplastic surgery This table lists validated instruments and imaging modalities utilized for objective evaluation in gynecoplastic surgery DIVA: day-to-day impact of vaginal aging; ICC: intraclass correlation coefficient

Instrument	Type	Parameters measured	Reference
Vaginal Health Index	Clinician-reported	Elasticity, fluid volume, pH, epithelial integrity, and moisture	Bachmann et al. [21]
Vulvar Health Index	Clinician-reported	Texture, elasticity, and pigmentation	Cucinella et al. [22]
Female Sexual Function Index	Patient-reported	Desire, arousal, lubrication, orgasm, and satisfaction	Rosen et al. [23]
DIVA questionnaire	Patient-reported	Impact of vaginal aging on daily life and sexual well-being	Huang et al. [24]
3D photogrammetry/stereophotogrammetry	Imaging	Labial volume, soft tissue contour, and reproducibility >90% ICC	Almadori et al. [25]
High-frequency/transperineal ultrasound	Imaging	Dermal thickness, fascial distance, and adipose atrophy	Montik et al. [26]

Technological Evolution and Regenerative Integration

Technological progress has significantly improved the precision and safety of female genital surgery. The advent of lasers and other energy-based technologies has enabled more controlled tissue interaction, superior hemostasis, and shorter recovery times compared with conventional methods. These innovations have expanded the therapeutic potential of genital procedures, allowing surgeons to address both functional and aesthetic goals with enhanced predictability and reduced complication rates. By promoting controlled collagen remodeling, accelerated healing, and improved mucosal quality, laser technology has contributed to the refinement and reproducibility of surgical outcomes. When employed within ethical and evidence-based frameworks, these advances exemplify how innovation strengthens patient safety and functional restoration in gynecoplastic surgery.

Educational and Ethical Implications

A recurring theme across the reviewed literature is the need for structured education, ethical oversight, and terminological clarity. Professional organizations have emphasized the importance of anatomical accuracy, informed consent, and avoidance of commercial bias. The adoption of the gynecoplastic surgery terminology provides a cohesive platform to guide formal training curricula, establish certification standards, and promote multidisciplinary collaboration. By merging surgical innovation with functional science and ethical governance, gynecoplastic surgery represents a natural evolution toward a standardized academic specialty capable of sustaining both scientific advancement and patient well-being (Table 3).

**Table 3:** Alignment between IUGA-AUGS (2022) recommendations and the gynecoplastic framework This table illustrates how the proposed gynecoplastic surgery framework addresses the ethical, terminological, and educational gaps identified by the IUGA and the AUGS (2022) [15] IUGA: International Urogynecological Association; AUGS: American Urogynecologic Society

IUGA-AUGS 2022 recommendation	Corresponding element in gynecoplastic surgery
Establish clear, standardized terminology for "cosmetic gynecology"	"Gynecoplastic Surgery" as a unified academic descriptor encompassing aesthetic, reconstructive, and functional goals
Emphasize patient education and informed consent	Integration of ethical transparency and patient comprehension as core components of gynecoplastic clinical practice
Develop training and competency guidelines	Structured curricula that include anatomy, energy-based technology, and regenerative techniques
Encourage multidisciplinary collaboration	Inclusion of gynecologists, plastic surgeons, dermatologists, and regenerative medicine specialists under one conceptual domain

Discussion

The proposal for gynecoplastic surgery as a unified academic terminology emerges from a critical need to standardize practices, outcomes, and education in female genital surgery [9,10,15]. The fragmentation of the field under various terminologies, such as cosmetic gynecology, aesthetic vulvar surgery, and functional gynecology, has inadvertently limited academic cohesion and ethical oversight. Our review indicates that the historical evolution of the field has created four distinct anatomical and procedural domains that, despite being addressed by different specialists (plastic surgery, gynecology, and urogynecology), share common goals: anatomical restoration, aesthetic harmony, and functional enhancement [16-19].

This unified terminology provides a scientifically coherent and ethically defensible identity for this unified discipline. By integrating the nomenclature of aesthetic refinement with the principles of reconstructive and functional surgery, it mirrors the successful consolidation seen in other interdisciplinary fields, notably oncoplastic breast surgery [4,5]. This model is ethically crucial as it frames all procedures, even those primarily sought for aesthetic reasons, within a medical context that mandates precise anatomical understanding, objective criteria for success, and avoidance of nonevidence-based claims [1-3].

In this context, it is essential to address how gynecoplastic surgery integrates with existing standards, such as the 2022 IUGA-AUGS Joint Report [15]. While the IUGA-AUGS consensus provides fundamental descriptive terms for specific procedures to eliminate commercial ambiguity, gynecoplastic surgery acts as a broader academic umbrella. It does not overlap with these technical descriptions but rather organizes them within a unified surgical philosophy. By providing this disciplinary structure, the framework serves as a safeguard against marketing-driven terminology, ensuring that the "cosmetic" aspects are always subordinated to reconstructive and functional medical principles, thus aligning with the cautionary stance of international regulatory bodies.

A core finding of this review is the increasing reliance on objective assessment tools. The adoption of validated indices like the VHI and VuHI, combined with volumetric imaging modalities, moves the field away from purely subjective patient satisfaction toward measurable outcomes [20]. This shift is essential for establishing evidence-based guidelines, which is a key requirement highlighted by professional organizations (ACOG, RCOG, FIGO) [1-3]. By standardizing evaluation, gynecoplastic surgery facilitates the creation of robust research protocols necessary to justify and validate new surgical and nonsurgical technologies.

The presented conceptual framework is constrained by the current literature's reliance on narrative reviews and descriptive studies, lacking extensive, long-term RCTs. Furthermore, the field remains controversial, often conflating patient autonomy with potential commercial exploitation [1-3,15]. Our proposed terminology does not resolve these ethical debates but provides a platform for addressing them within a structured academic discipline, ensuring that ethical governance is central to training and practice. The term gynecoplastic surgery aims to elevate the discussion above the aesthetic-versus-functional dichotomy to a focus on integrated health and well-being.

## Conclusions

The transformation of female genital surgery from isolated aesthetic or reconstructive procedures into an integrated, scientifically grounded discipline reflects the maturation of an entire field. The term gynecoplastic surgery embodies this evolution: it is not a marketing label, but the academic identity of a surgical philosophy that unites anatomy, function, and aesthetics under the governance of ethics and evidence.

The formal adoption of this terminology will provide the structure needed to educate future surgeons, standardize outcomes, and safeguard patient welfare. It will draw clear boundaries between medicine and commerce, replacing ambiguity with precision and regulation. Just as oncoplastic surgery once redefined breast reconstruction through unity of purpose, gynecoplastic surgery now stands to redefine female genital surgery through science, responsibility, and excellence.

## References

[1] Syed SA, Anwar A (2025). The globally rising tide of cosmetic gynaecology: are providers aware of the ethical aspects?. Cureus.

[2] (2025). Ethical considerations in relation to female genital cosmetic surgery. https://qna.files.parliament.uk/qna-attachments/788291/original/HL3083%20B.pdf?utm_medium=email&utm_source=transaction.

[3] (2025). Elective female genital cosmetic surgery. ACOG Committee on Gynecologic Practice. https://www.acog.org/clinical/clinical-guidance/committee-opinion/articles/2020/01/elective-female-genital-cosmetic-surgery?utm_medium=email&utm_source=transaction.

[4] International Federation of Gynecology and Obstetrics (FIGO (2025). International Federation of Gynecology and Obstetrics (FIGO). Ethical concerns in female cosmetic genital surgery. https://www.figo.org/news/ethical-concerns-female-cosmetic-genital-surgery.

[5] Audretsch W (1994). Onco-plastic surgery: “target” volume reduction (BCT-mastopexy), lumpectomy reconstruction (BCT-reconstruction) and flap-supported operability in breast cancer. Proceedings of the 2nd European Congress on Senology.

[6] Clough KB, Lewis JS, Couturaud B, Fitoussi A, Nos C, Falcou MC (2003). Oncoplastic techniques allow extensive resections for breast-conserving therapy of breast carcinomas. Ann Surg.

[7] Kaufman CS (2019). Increasing role of oncoplastic surgery for breast cancer. Curr Oncol Rep.

[8] Munhoz AM, Aldrighi CM, Ferreira MC (2007). Paradigms in oncoplastic breast surgery: a careful assessment of the oncological need and esthetic objective. Breast J.

[9] Goodman MP (2011). Female genital cosmetic and plastic surgery: a review. J Sex Med.

[10] Iglesia CB, Yurteri-Kaplan L, Alinsod R (2013). Female genital cosmetic surgery: a review of techniques and outcomes. Int Urogynecol J.

[11] Hamori CA (2014). Aesthetic surgery of the female genitalia: labiaplasty and beyond. Plast Reconstr Surg.

[12] Sorice SC, Li AY, Canales FL, Furnas HJ (2017). Why women request labiaplasty. Plast Reconstr Surg.

[13] Clerico C, Lari A, Mojallal A, Boucher F (2017). Anatomy and aesthetics of the labia minora: the ideal vulva?. Aesthetic Plast Surg.

[14] Placik OJ, Devgan LL (2019). Female genital and vaginal plastic surgery: an overview. Plast Reconstr Surg.

[15] Developed by the Joint Writing Group of the International Urogynecological Association and the American Urogynecologic Society (2022). Joint report on terminology for cosmetic gynecology. Int Urogynecol J.

[16] El-Khatib H (2011). Mons pubis ptosis: classification and strategy for treatment. Aesthetic Plast Surg.

[17] Fasola E, Gazzola R (2016). Labia majora augmentation with hyaluronic acid filler: technique and results. Aesthet Surg J.

[18] Palacios S (2018). Vaginal hyperlaxity syndrome: a new concept and challenge. Gynecol Endocrinol.

[19] González-Isaza P, Sánchez-Borrego R (2024). Topographic Labiaplasty: From Theory to Clinical Practice. Amolca.

[20] Rodas A, Sanabria G, González-Isaza P (2025). Narrative review of clinical indices and imaging tools, with a proposal for an Integrative Grading Scale for vulvovaginal aging. Cureus.

[21] Bachmann GA, Notelovitz M, Kelly SJ, Owens A, Thompson C (1992). Long-term non-hormonal treatment of vaginal dryness. Clin Pract Sexuality.

[22] Cucinella L, Tiranini L, Cassani C (2024). Insights into the vulvar component of the genitourinary syndrome of menopause (GSM). Maturitas.

[23] Rosen R, Brown C, Heiman J (2000). The Female Sexual Function Index (FSFI): a multidimensional self-report instrument for the assessment of female sexual function. J Sex Marital Ther.

[24] Huang AJ, Gregorich SE, Kuppermann M (2015). Day-to-Day Impact of Vaginal Aging questionnaire: a multidimensional measure of the impact of vaginal symptoms on functioning and well-being in postmenopausal women. Menopause.

[25] Almadori A, Speiser S, Ashby I, Lacher R, Bishop D, Mosahebi A, Butler PE (2022). Portable three-dimensional imaging to monitor small volume enhancement in face, vulva, and hand: a comparative study. J Plast Reconstr Aesthet Surg.

[26] Montik N, Grelloni C, Delli Carpini G, Petrucci J, Di Giuseppe J, Ciavattini A (2025). Transperineal vulvar ultrasound: a review of normal and abnormal findings with a proposed standardized methodology. Diagnostics (Basel).

